# Molecular diversity and distribution of marine fungi across 130 European environmental samples

**DOI:** 10.1098/rspb.2015.2243

**Published:** 2015-11-22

**Authors:** Thomas A. Richards, Guy Leonard, Frédéric Mahé, Javier del Campo, Sarah Romac, Meredith D. M. Jones, Finlay Maguire, Micah Dunthorn, Colomban De Vargas, Ramon Massana, Aurélie Chambouvet

**Affiliations:** 1 Biosciences, University of Exeter, Geoffrey Pope Building, Exeter EX4 4QD, UK; 2 Canadian Institute for Advanced Research, CIFAR Program in Integrated Microbial Biodiversity, Toronto, Ontario, Canada M5G 1Z8; 3 CNRS, UMR 7144, EPEP—Évolution des Protistes et des Écosystèmes Pélagiques, Station Biologique de Roscoff, Roscoff 29680, France; 4 Department of Ecology, University of Kaiserslautern, 67663 Kaiserslautern, Germany; 5 Department of Botany, University of British Columbia, 3529-6270 University Boulevard, Vancouver, British Columbia, Canada V6T 1Z4; 6 Department of Life Sciences, Natural History Museum, Cromwell Road, London SW7 5BD, UK; 7 Genetics, Evolution and Environment, University College London, London WC1E 6BT, UK; 8 Department of Marine Biology and Oceanography, Institut de Ciències del Mar (CSIC), Barcelona, Catalonia, Spain

**Keywords:** 454 pyrosequencing, chytrids, Dikarya, sediment communities

## Abstract

Environmental DNA and culture-based analyses have suggested that fungi are present in low diversity and in low abundance in many marine environments, especially in the upper water column. Here, we use a dual approach involving high-throughput diversity tag sequencing from both DNA and RNA templates and fluorescent cell counts to evaluate the diversity and relative abundance of fungi across marine samples taken from six European near-shore sites. We removed very rare fungal operational taxonomic units (OTUs) selecting only OTUs recovered from multiple samples for a detailed analysis. This approach identified a set of 71 fungal ‘OTU clusters' that account for 66% of all the sequences assigned to the Fungi. Phylogenetic analyses demonstrated that this diversity includes a significant number of chytrid-like lineages that had not been previously described, indicating that the marine environment encompasses a number of zoosporic fungi that are new to taxonomic inventories. Using the sequence datasets, we identified cases where fungal OTUs were sampled across multiple geographical sites and between different sampling depths. This was especially clear in one relatively abundant and diverse phylogroup tentatively named Novel Chytrid-Like-Clade 1 (NCLC1). For comparison, a subset of the water column samples was also investigated using fluorescent microscopy to examine the abundance of eukaryotes with chitin cell walls. Comparisons of relative abundance of RNA-derived fungal tag sequences and chitin cell-wall counts demonstrate that fungi constitute a low fraction of the eukaryotic community in these water column samples. Taken together, these results demonstrate the phylogenetic position and environmental distribution of 71 lineages, improving our understanding of the diversity and abundance of fungi in marine environments.

## 1. Background

Fungi are osmotrophs and therefore feed by secreting enzymes into the environment to process nutrients externally before taking the resulting metabolites into the cell [1–3]. Using this strategy, Fungi have diversified into important parasitic, mutualistic and saprotrophic forms [2]. Fungi are particularly diverse and abundant in soils, plant-associated habitats [4–11] and freshwater environments [12–14]. However, the diversity and abundance of fungal microbes in marine environments are unclear, although recent progress has documented 1112 putative marine fungi [15]. Culture/morphology-based analyses have recovered fungi from marine samples [16,17], yet the diversity recovered is much lower than that of terrestrial environments. For example, Kis-Papo [18] reported 467 marine species of fungi from 244 genera, while Hyde *et al.* [19] report 444 species, both results are equivalent to less than 1% of described fungal species at the time of these analyses.

Polymerase chain reactions (PCR) that target the eukaryotic small subunit ribosomal RNA (SSU rRNA) gene have shown a low recovery of fungal sequences from the upper marine water column of both coastal and open water environments [20,21]. Meta-analyses of marine water column samples including 23 coastal libraries (1349 clones) and 12 open-water libraries (826 clones) recovered 16 fungal clones (0.8%) [21], suggesting that fungi are present in low diversity and abundance in water column environments or the methodologies used are biased against recovery of fungal sequences. The low representation of fungi in marine water column clone library analyses is in contrast to equivalent freshwater analyses that demonstrate both a high diversity and relative abundance of fungal OTUs [12–14].

The PCR with primers that preferentially amplify fungal SSU rRNA genes has recovered additional diversity from marine sediment, anoxic and deep-water samples [22–24]. Many of the sequences recovered constitute closely related groups sampled across different environments [25]. Meta-analysis has also demonstrated that clone library sampling of marine fungi was in the most part dominated by Dikarya, yet contained a significant diversity of sequences that branch close to chytrids (fungi with a flagellated spore). Furthermore, this ‘chytrid-like’ diversity encompassed highly variant rDNA sequences when compared with sequences from described fungi [25]. This marine diversity of chytrid-like phylotypes also includes several SSU sequences that branched with the Cryptomycota (syn. Rozellida/Rozellomycota) [25,26], a diverse putative phylum that includes the intracellular myco-parasite *Rozella* and is thought to group with microsporidia as the deepest branch in the Fungi or sister to the Fungi [27–29].

In contrast to the surface marine water column studies, clone library studies using DNA recovered from deep-sea environments have identified a higher relative representation of fungal sequences [30–32]. Both second-generation SSU V4 rR/DNA diversity tag sequencing [33] and meta-transcriptome sequencing [34] suggest fungi dominate eukaryotic communities in deep-sea sediments. Edgcomb *et al.* targeted the eukaryotic community of sediment cores using both RNA and DNA templates and demonstrated that the diversity recovered was dominated by fungi, specifically basidiomycete yeasts branching close to *Cryptococcus* and *Malassezia* [31] and similar results have been recovered in additional studies [23,30–32,35]. Furthermore, fungi have also been recovered from marine animals, algae, muds and hydrothermal vents [16,18,19,36,37]. Here, we use BioMarKs V4 SSU rR/DNA derived Roche/454 sequence tag dataset [38] from 130 samples from six European marine sites combined with fluorescence microscopy for the detection of eukaryotic microbes with chitin cell walls to investigate the diversity and abundance of fungi in water column and surface sediment samples.

## 2. Results and discussion

### (a) Sampling of multi-provenance operational taxonomic unit-clusters

**Figure 1.**
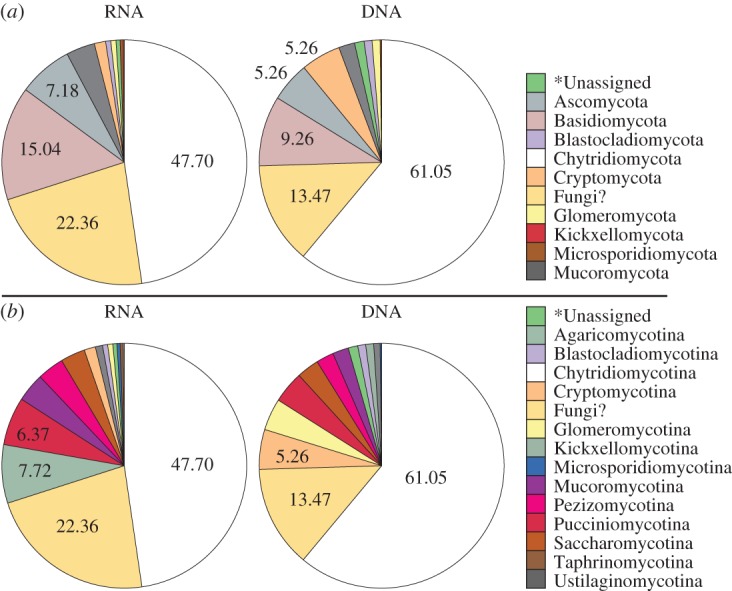
Taxonomic composition of the fungal BioMarKs sequences prior to multi-occurrence filtering. (*a*) Phylum-level groupings. (*b*) Subdivision level groupings. ‘*Unassigned’ when taxonomy could not be assigned using the higher support threshold used here. ‘Fungi?’ means the sequences can be assigned to fungi but not to a phylum or subdivision.

Initial clustering of the tag sequences identified 1752 fungal SWARM-OTUs encompassing 10 840 reads from European marine waters (table 1). Figure 1 summarizes the taxonomic assignment of these 1752 fungal SWARM-OTUs. Taxonomic assignment was accomplished by using the most numerous sequence read within each SWARM-OTU for VSEARCH against a copy of the PR2 V4 SSU rRNA database [39]. The fungal-assigned OTUs recovered were dominated by Chytridiomycota and unclassified fungi followed by Ascomycota and Basidiomycota.

**Table 1.** Summary of sequencing results.

sequencing results	number of reads
454 reads included in the analysis (‘cleaned’)	1 431 325
SWARM-OTUs classified as fungi (before multi-occurrence threshold rule applied)	10 840
processed^a^ fungal reads	7202
total reads from sediment samples (*n* = 24)	216 013
processed^a^ fungal reads from sediment samples	5249
total reads from water column samples (*n* = 106)	1 215 312
processed^a^ fungal reads from water column	1955

^a^After multi-occurrence threshold rule applied.

These reads were filtered in two additional ways: first, representative sequences from each OTU were aligned and masked using the approaches described in the electronic supplement material. This allowed for manual checks to identify sequencing errors such as homo-polymer errors. Phylogenetic analyses demonstrated many OTUs were phylogenetically identical when placed in trees generated from masked alignments. Therefore, the masked OTUs were re-clustered using CD-HIT, allowing for 1 nt variation, to form ‘OTU clusters'.

Many of the BioMarKs sampling sites were close to land, as such the diversity profile sampled is likely to be influenced by passive dispersal of spores and other propagules from terrestrial environments. To minimize this source of artefact, and to remove OTUs that were represented by a low number of sequences, we retained only those OTU clusters present in two or more samples if one sample was derived from an RNA template, or present in three or more samples if the OTU clusters were present only in DNA samples. This filtering process resulted in 71 OTU clusters encompassing 7202 reads (table 1) compared with OTUs initially identified (1752 OTUs, 10 840 reads). Although this strict filtering process removed 96% of the diversity of OTUs, it retained 66% of the reads initially assigned to fungi. Indeed, 1107 (77%) of the marine fungal OTUs removed because of low recovery across samples were single sequence-single sample OTU clusters. Furthermore, only 34 OTUs were excluded because they were present in two DNA samples. These 34 ‘DNA OTUs' encompassed 170 sequences (0.01% of the total quality checked sequencing effort). It is possible that these criteria may lead to erroneous exclusion of true marine fungal OTUs, but these low numbers suggest that this is a minor factor, and unimportant in comparison to other sources of artefact such as partial primer coverage of the Fungi and/or incomplete sequence sample saturation of the amplicon libraries. However, such processing allows us to identify a subset of fungi likely to be functional in these marine environments.

The 71 ‘OTU clusters' contained an average of 99.7% (±s.e.m. = 0.106) sequence similarity (comparison of unmasked sequences reads) with 99.3% being the lowest mean pair-wise level of similarity within a cluster (±s.e.m. = 0.106; electronic supplementary material, table S3). Nonetheless, each OTU cluster potentially encompasses considerable strain/species variation. This is because the V4 SSU rDNA, as with all regions of the SSU rRNA encoding gene, does not encompass enough variation to accurately track species diversity in the Fungi (as such ITS markers are often favoured [14,40]). Consequently, the 71 OTU clusters identified are likely to represent clusters of closely related strains/species.

### (b) Diversity of repeat-sampled operational taxonomic units

**Figure 2.**
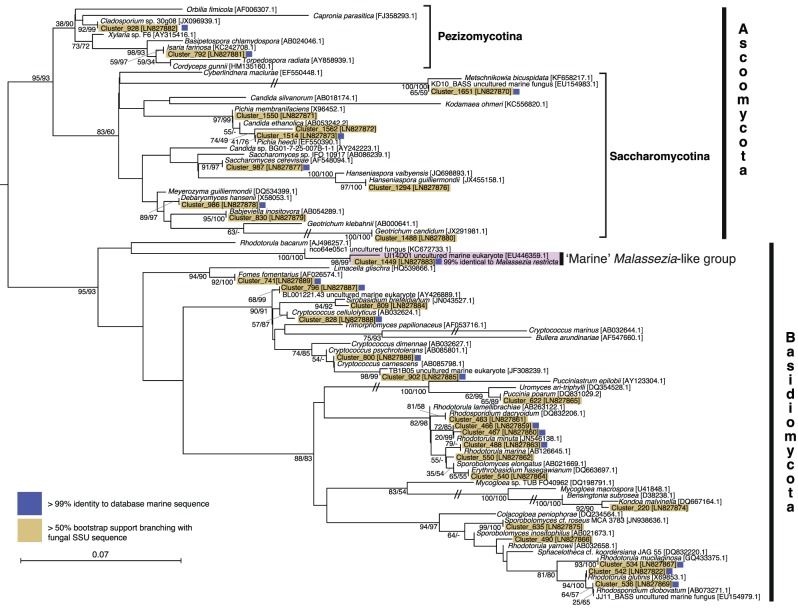
Phylogeny of the Dikarya marine OTU clusters. ML phylogeny calculated from an alignment of 94 sequences and 342 positions. Bootstrap values from both 1000 ML and 1000 Log-Det distance pseudo-replicates are shown when >50%. Branches with a double slash mark indicate a branch reduced in length by 1/2. Blue squares next to each sequence indicates OTU clusters which have >99% identity to a database sequence from a marine environment.

**Figure 3.**
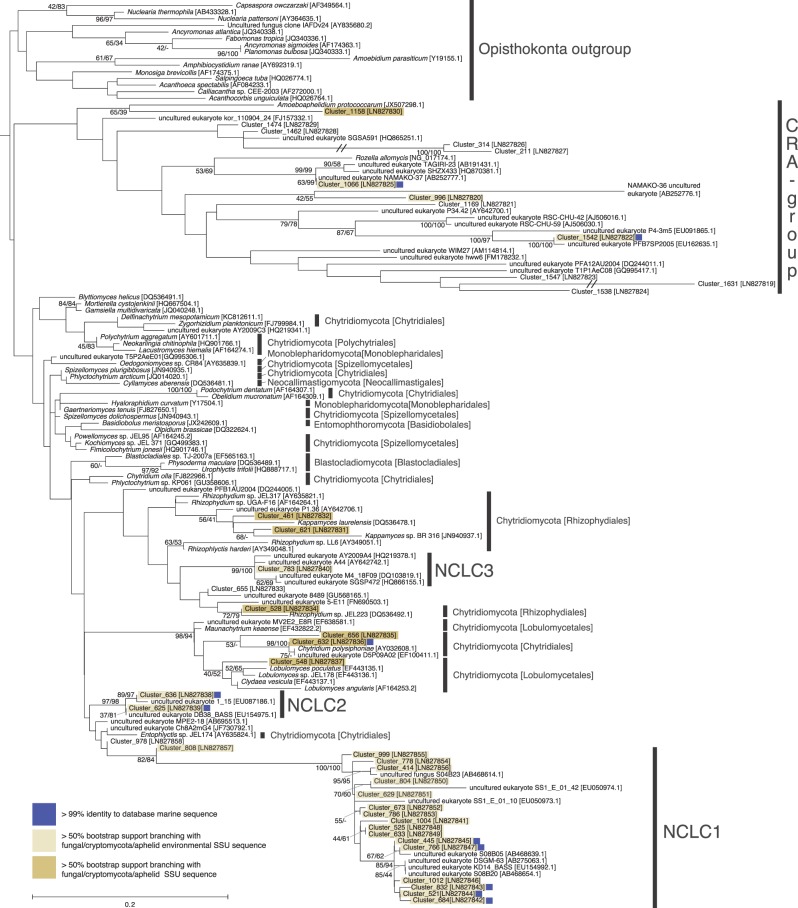
Phylogeny of the chytrid-like marine OTU clusters. ML phylogeny calculated from an alignment of 136 sequences and 316 positions. Bootstrap values from both 1000 ML and 1000 Log-Det distance pseudo-replicates are shown when the values are greater than or equal to 50%. Blue squares next to each sequence indicates OTU clusters that have greater than 99% identity to a database sequence from a marine environment. Branches with a double slash mark indicate a branch reduced in length by 1/2. ‘CRA group’ shortened name given to Cryptomycota/Rozell[ida]-omycota/Aphelid group. NCLC (Novel Chytrid-Like-Clade) groups are labelled.

Seventy-one rDNA sequences, each one representing an OTU cluster, were aligned with sequences derived from known fungal taxa and environmental sequences. Phylogenetic analysis allowed us to assign these sequences to two separate alignments: Dikarya (31 OTU clusters; figure 2) or chytrid-like (40 OTU clusters; figure 3). Phylogenetic analyses were then conducted using both maximum-likelihood and Log-Det distance methods. These analyses placed all 31 Dikarya-like sequences and seven chytrid-like sequences with known fungal or Cryptomycota/Rozell[ida]-omycota/Aphelid CRA (CRA) species with greater than or equal to 50% bootstrap support according to one or both phylogenetic methods. Twenty-three of the chytrid-like SSU rDNA sequences branch with greater than or equal to 50% bootstrap support with published environmental SSU rDNA sequences that had previously been shown to branch within the Fungi/CRA sequences ([12,13,23,41,42]; figure 3) using full-length SSU rDNA phylogenies. The 10 remaining sequences affiliated with chytrid-like sequences (eight specifically with CRA) but their phylogenetic placement was not supported by greater than or equal to 50% bootstrap support. Fifty per cent is a low level of bootstrap support for identifying phylogenetic affiliations; it was used here as the phylogenies are calculated from short sections of the SSU rRNA encoding gene with relatively few positions sampled for the alignment (i.e. 342 and 316). As such phylogenetic analysis of these datasets is unlikely to consistently identify strong bootstrap results for even established phylogenetic relationships.

#### (i) Dikarya diversity

A diversity of Dikarya phylotypes was detected, such as *Rhodotorula*, *Rhodosporidium*, *Sporobolomyces*, *Kondoa* and *Cryptococcus* (Basidiomycota yeasts), and *Geotrichum*, *Debaryomyces*, *Saccharomyces*, *Candida* and *Pichia* (Ascomycota yeasts). The sequences sampled also include an OTU cluster representative of the marine *Malassezia*-like yeast [35]. These results are consistent with previous findings that the marine Dikarya is dominated by lineages capable of living as yeasts [23,25]. Possible alternative explanations for this result could be an experimental artefact arising from filtration and/or DNA/RNA extraction methods that do not adequately recover template from filamentous fungi with robust cell walls (i.e. Pezizomycotina), consistent with this hypothesis, very few Pezizomycotina (2.32 and 3.52%) were recovered in the 454 sequences prior to multi-occurrence filtering (figure 1*b*).

#### (ii) Chytrid diversity

**Figure 4.**
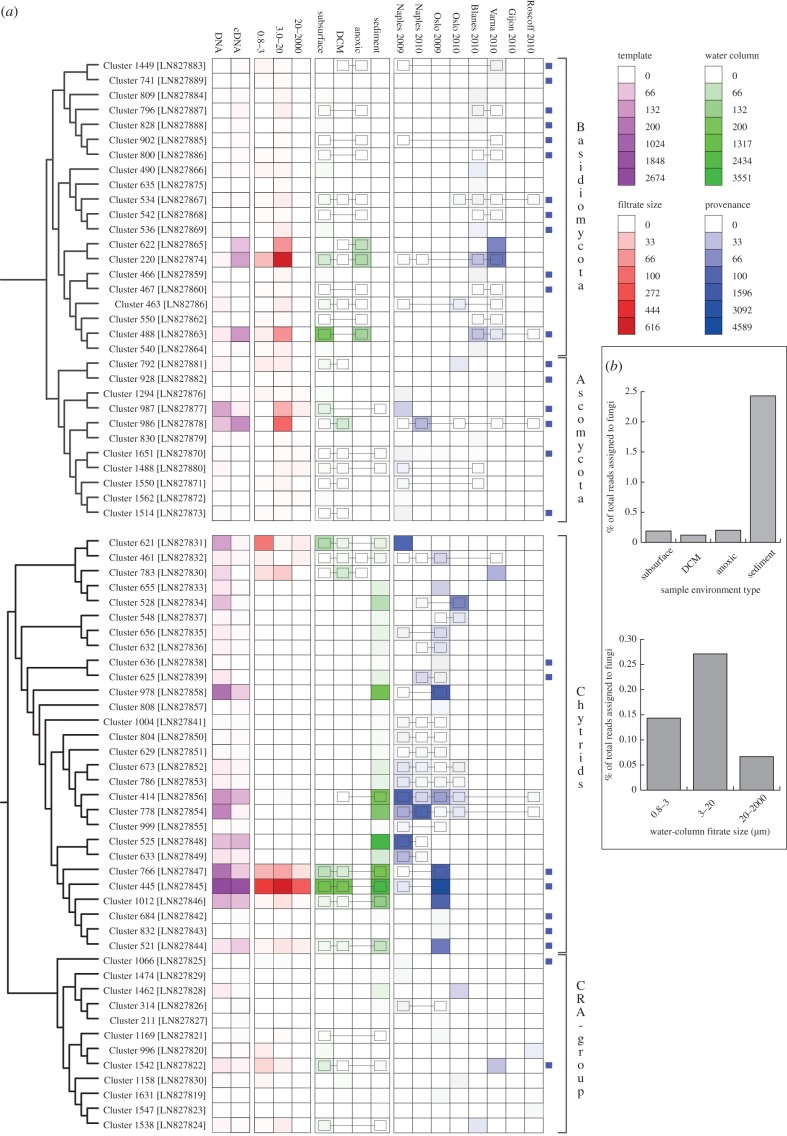
(*a*) Heat maps showing the sampling provenance of the 71 OTU clusters. Blue squares at the end of a row indicate OTU clusters that have greater than 99% identity to a database sequence from a marine environment. Colour scales are detailed in the legend box (indicating number of reads in each case). The linked boxes on the heat map indicate connected OTU clusters across sampling sites. (*b*) Per cent of sequencing effort assigned to fungi from different environment types. These results are calculated after the multi-occurrence threshold rule is applied and show a clear increase in fungal representation in sediment environments, either because fungal diversity and abundance is increased in this environment type or because the nucleotide extraction protocol differed between water column and sediment samples.

The tag sequencing recovered a diversity of chytrid-like sequences (figure 3). Six OTU clusters branched with known Chytridiomycota, e.g. *Lobulomyces*, *Chytridium* (a close relative of *C. polysiphonia* a parasite of algae [43]) and *Kappamyces*. These data also demonstrate 20 OTU clusters branching close to chytrid-like environmental DNA sequences. Seventeen of these OTU clusters branched in a clade defined by a long terminal branch and bootstrap support of 82/84%, and encompassing a diversity of shorter branches, named here ‘Novel Chytrid-Like-Clade 1’ (NCLC1, figure 3). This phylogenetic grouping had previously been identified as a divergent marine clade representing a ‘basal’ branch of fungi [23–25,44]. Indeed, this clade was named Basal Clade 1 by Nagahama *et al.* [44]. Six NCLC1 OTU clusters (414, 778, 766, 445, 1012 and 521) showed a high relative representation in both DNA- and RNA-derived libraries (figure 4*a*). Furthermore, OTU cluster group 445 was recovered in all the filtration size fractions, suggesting it has a multimodal life cycle as both a small (e.g. spores/cysts) and large cellular form (e.g. multi-cellular [zoo]sporangia or forming saprotrophic or symbiotic interactions). The phylogenetic data presented in figure 3 demonstrate two additional clades labelled NCLC2 and NCLC3 that include chytrid-like environmental phylotypes recovered from aquatic environments.

The phylogenetic results also demonstrate a diversity of 12 OTU clusters that branch with the CRA group (figure 3). This is consistent with previous data suggesting that representatives of this group are present in marine environments [26], although the OTU clusters identified were recovered at a low relative proportion of the sequences (figure 4*a*). The phylogenetic analysis recovered four OTU clusters branching with the CRA group with greater than or equal to 50% bootstrap support (Clusters: 1542, 996, 1066, 1158). Of interest, Cluster 1066 is part of a putative marine/halotolerant branch [41,45–47]. Cluster 1542 is closely related to sequences sampled from marine and freshwater environments [48–50] and Cluster 1158 is a divergent relative of *Amoeboaphelidium* [51]. These data show that the majority of the basally derived fungal lineages detected in these environments belong to Chytridiomycota lineages and not to the CRA group.

### (c) Biogeographic distribution of operational taxonomic unit clusters

Five of the Dikarya OTU clusters show biogeographic distribution patterns across three or more geographical sampling sites (figure 4*a*, OTU clusters: 534 (*Rhodotorula mucilaginosa*), 986 (*Debaromyces hansenii*), 463 (*Rhodosporidium dacryoidum*), 220 (similar to *Kondoa malvinella*) and 488 (*Rhodotorula marina*)). Notably, two of these OTUs (220 and 488) were highly represented in the Varna Black Sea anoxic environment while also showing distribution patterns across multiple geographical sites (figure 4*a*).

Eight of the chytrid-like sequences were recovered from three or more geographical sites (OTU clusters: 461, 1004, 804, 629, 673, 786, 414, 778), demonstrating a high degree of distribution for these lineages. Interestingly, seven of these OTUs branch within the NCLC1 group. This group has also been detected in previous marine environmental DNA clone library analyses including hydrothermal vent samples [23–25,44]. This pattern of sequence recovery is consistent with the conclusion that NCLC1 encompasses a significant marine radiation of fungi. None of the CRA group OTU clusters were represented in three or more geographical sites.

Notably, 18 of the 31 Dikarya and 10 of the 41 chytrid OTU clusters showed greater than 99% sequence similarity to an SSU rDNA phylotype (figures 2, 3 and 4*a*) previously sampled from the marine environment [25]. These results further demonstrate evidence of the biogeographic distribution patterns of the fungal OTU clusters identified here (figure 4*a*) and provide additional support for the hypothesis that these groups represent *bona fide* marine lineages.

The sequence data demonstrated a higher recovery of fungi sequences from sediment compared with water column (figure 4*b*), suggesting fungal diversity/abundance is increased in the sediment. This is consistent with a high abundance and diversity of fungi generally found in solid substrate detrital environments, i.e. soils [8] and aquatic sediments [31,33]. However, this observation needs further experimental validation as comparisons between the water column and sediment samples are limited here because DNA and RNA recovery were not conducted using equivalent nucleotide extraction processes (see the electronic supplementary material) and so it is possible that water column sampling failed to recover fungal species with robust cell walls. This could explain the reduced recovery of fungal diversity from water column samples generally (figure 4*b*) and specifically fungi from the 20 to 2000 µm size fraction where the filamentous fungal forms, with robust and refractory cell-wall structures, are likely to be sampled. Indeed, taxonomic assignment analysis of the total BioMarKs fungal-assigned dataset showed that a very low proportion of the total sequences was assigned to fungal groups known to form filamentous structures in terrestrial environments, for example Pezizomycotina represented only 2.32% and 3.52% of the total DNA and RNA reads, respectively (figure 1*b*). It also could explain why Dikarya yeasts and chytrid-like sequences were preferentially recovered in these datasets, as both cellular forms are relatively fragile and therefore more readily sampled for RNA/DNA sequencing.

### (d) Chitin-walled cell counts in water column samples

Detection of cells with a chitin wall using the stain Calcofluor White (CFW) has been proposed as a method for assessment of abundance of fungi in water column samples [52]. This method is problematic as many non-fungal species have chitin on their cell surface [53–56], some fungal life cycle stages do not have chitin cell walls (e.g. zoospores) and furthermore, CFW binds to other cell surface polysaccharides such as cellulose [57,58]. We have adapted this approach replacing CFW with a fluorescent-labelled wheatgerm agglutinin (WGA) [27] lectin, which binds chitin. WGA can bind other polymers containing *n*-acetylglucosamine, specifically bacterial peptidoglycan in Gram-positive bacteria, as such it is important to co-stain with a second marker to confirm the target cell is a eukaryote. Here we used DAPI (4′,6-diamidino-2-phenylindole) to confirm the target cell contained a distinct DNA containing nucleus-like structure [59,60].

Initially, to compare CFW and WGA approaches we used a separate sample, with a high abundance of chitin cell-walled microbes, to investigate the relative abundance of WGA-stained cells and of cells stained with both CFW and WGA. Counting three independent filters demonstrated a concentration of 1248 cells ml^−1^ (s.d. ±232 cells) that had double cell-wall staining (WGA and CFW), while for single WGA staining we observed 1231 cells ml^−1^ (±580). These results suggest that WGA performs similar to the double-staining approach.

**Figure 5.**
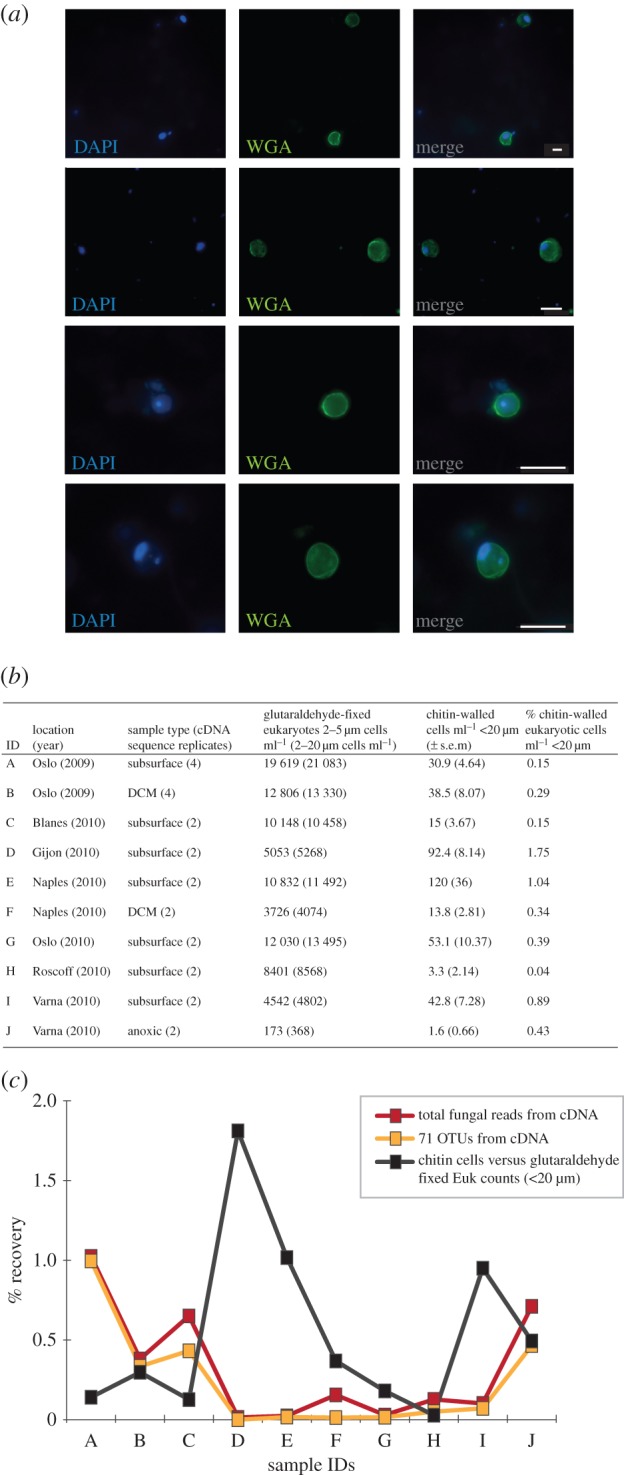
The abundance of fungal cells in the marine water column samples using relative abundance in RNA-derived tag libraries and chitin cell-wall detection. (*a*) Examples of chitin-walled cells detected using wheatgerm agglutinin (WGA) and DAPI detection. All scale bars measure 5 µm. (*b*) Provenance and abundance of eukaryotic cells and eukaryotic cells with a chitin cell wall. (*c*) Comparison of per cent recovery of fungal sequences from RNA sequencing to per cent chitin cells across 10 samples (for sample IDs, see figure 5*b*).

As part of the BioMarKs sampling strategy, microbial cells were collected and processed for fluorescent microscopy from the same environmental samples as used for the DNA/RNA samples. Using 10 representative samples, we used microscopy to identify microbes with chitin cell walls (figure 5*a*) and counted the total number of eukaryotic cells per millilitre recovered in the less than 20 µm filtration fractions that had a WGA-labelled wall. The WGA microscopy confirmed the presence of spherical cells enclosed within chitin walls with a distinct DAPI-stained nucleus-like structure, i.e. putative yeast or encysted cells (figure 5*a*), but very few cells with filamentous structures indicative of hypha were identified (less than 50; electronic supplementary material figure S1). These results are consistent with the filtration size fraction (less than 20 µm), i.e. it is unlikely that we would sample fungal hyphal cells at this size fraction.

The 10 samples contained a mean of 1–120 eukaryotic cells with putative chitin walls per millilitre (figure 5*b*). These results were compared with the total eukaryotic cell counts per millilitre from the glutaraldehyde-fixed samples [61]. This demonstrated between 0.15 and 1.75% of the eukaryotic cells in the water column possessed a chitin cell wall (figure 5*b*). This low rate of recovery is consistent with the RNA relative abundance data from equivalent samples, which demonstrates fungal tag sequences represent a small proportion of the sequencing results recovered (figure 5*c*) and confirms that there is no abundant population of fungal cells (less than 20 µm) with chitin walls in the water column that were not detected as part of the molecular sampling. Although the RNA and cell counting results are similar in terms of low proportional representation of putative fungi, these data show a weak correlation between parallel samples (figure 5*c*, *R*^2^ = 0.2186, *p* = 0.12). This weak correlation suggests that relative RNA tag abundance and/or chitin detection is a bad proxy for identifying low abundance populations of fungi in the water column.

## 3. Conclusion

Eukaryotic diversity tag sequencing from European water column and sediment samples processed to identify repeat-sampled OTUs demonstrates a low diversity of repeat-sampled putative marine fungi. Furthermore, the RNA-derived tag sequencing also suggests a low relative abundance of fungi (figure 5*c*). Cell-wall staining confirmed a low abundance of chitin-walled cells in representative water column samples including, but not exclusively, fungal cysts and yeast cells.

We applied a strict criterion for retaining OTU clusters present in multiple sample sets, a process that considerably reduced the number of OTU clusters by 96% but retained 66% of the sequence reads identified as fungi. We argue that this approach is valid as it allows us to identify OTUs that are likely to represent *bona fide* marine lineages and exclude OTUs with low representation across samples. Consistent with this approach, 28 of the 71 OTU clusters are greater than 99% identical to lineages previously sampled from marine environments (figures 2, 3 and 4*a*). Interestingly, these results demonstrate a substantial diversity of chytrid-like sequences that represent undescribed taxonomic groups, many of which occupy a distinct phylogenetic placement and encompass considerable diversity (e.g. NCLC1, figure 3).

The fungal OTU clusters identified were predominately chytrid-like and yeast Dikarya phylotypes. As discussed this profile may be a product of the sampling strategy. Alternatively, it may suggest that filamentous fungal forms such as Pezizomycotina are less suited for marine water column environments—instead preferentially colonizing solid substrates rich in organic matter such as soils and sediments [25]. As environmental DNA/RNA sampling increases, cross-comparisons will allow for an improved understanding of which OTU clusters represent true marine fungi. It is certain that increased sampling of different marine habitats, including, for example, animals and algae, would reveal further fungal diversity not captured in these samples. Future questions relating to the status of ‘marine’ fungi include: what are the ecological characteristics of the marine fungi that allow them to survive in these habitats, how frequently has the marine/terrestrial transition occurred, what are the trophic strategies employed by marine fungi (e.g. parasitism, saprotrophy or mutualistic symbiosis) [25]? However, many of the fungi identified here are likely to be difficult to propagate in culture, either because they are outgrown by contaminating terrestrial fungi also present in the environmental samples, or alternatively their life cycle is dependent on a symbiotic interaction. As such, targeted single cell genomics/transcriptomics [62] represents a useful tool for sampling marine fungi.

## Data accessibility

Electronic supplementary material that accompanies the online version of this article includes materials and methods, a description of the environments sampled (electronic supplementary material, tables S1 and S2), and a table showing the per cent similarity within each of the OTU clusters (electronic supplementary material, table S3). Electronic supplementary material, figure S1 eukaryotic cells with putative chitin cell walls and filamentous cell structures. Representative sequences of the 71 have been submitted to the European Nucleotide Archive: LN827819-LN827889. Additional supporting data are available at GitHub (https://github.com/guyleonard/marine_fungi with doi:10.5281/zenodo.16817). These data include: (i) all the sequences grouped into the 71 OTU clusters (fasta files), (ii) a spread-sheet showing the recovery of tag sequences classified in the 71 OTU clusters from the 130 environmental samples, (iii) the water column cell counting data, (iv) the two V4 SSU rDNA alignments as MASE files with mask details available using SeaView [63], and (v) the tree files of the phylogenies shown in figures 1 and 2.
